# The effect of mind-body exercise on cervical spine mobility of people with neck discomfort: A systemic review and meta-analysis of randomised controlled trials

**DOI:** 10.1371/journal.pone.0262429

**Published:** 2022-01-21

**Authors:** Xianhui Liao, Hao Chen, Beihai Ge

**Affiliations:** 1 Department of Sports, Wuhan EQ & IQ School, Wuhan, Hubei, China; 2 Institute of Physical Education and International Equestrian, Wuhan Business University, Wuhan, Hubei, China; 3 Department of Neurology, Guangxi Zhuang Autonomous Region Brain Hospital, Liuzhou, Guangxi, China; University of Mississippi Medical Center, UNITED STATES

## Abstract

**Background:**

Long-term and high-intensity work can lead to considerable discomfort in people’s cervical spines.

**Objectives:**

This study sought to explore the effect of mind-body exercise intervention on the cervical spine mobility of people with neck discomfort through meta-analysis.

**Methods:**

This study’s researchers were searched a total of five research databases for data retrieval: China National Knowledge Infrastructure (from 1979), Web of Science (from 1950), PubMed (from 1965), Cochrane (from 1991), and EBSCO (from 1949) (Date of retrieval: March 10, 2021). Two authors independently searched literature records, scanned titles, abstracts, and full texts, collected data, and assessed materials for risk of bias. Stata14.0 software was used for the data analysis (Registration number: INPLASY202140126).

**Results:**

Four articles were finally included with a total of 208 participants, and their age range was 18–65 years old. (1) Mind-body exercise intervention had a significant improving effect on Cervical extension, effect size of [SMD = 0.51 (95% CI 0.13 to 0.88), p <0.01; I2 = 45.2%], there was moderate heterogeneity; Mind-body exercise intervention had a significant improving effect on Cervical flexion, effect size of [SMD = 0.61 (95% CI 0.32 to 0.90), p <0.01; I2 = 5.7%], no heterogeneity; (2) Mind-body exercise intervention was no effect on the other four cervical range of motions; (3) The difference in participant’s neck discomfort was the source of heterogeneity, and all results had the potential risk of publication bias.

**Conclusion:**

This study showed that mind-body exercise had a positive effect on the extension and flexion of people with neck discomfort. However, further research and more reliable evidence were needed to prove that mind–body exercise could be used for the treatment of neck discomfort.

## Introduction

With the development of the economy and society, the pace of in-person work has gradually accelerated, resulting in longer and more intense work hours. The working methods of modern society are primarily different from the past due to electronic products. Long-term and high-intensity work can lead to considerable discomfort in people’s cervical spines [1].

Neck discomfort is a common symptom of students and office workers and other people that can lead to clear dysfunction in neck movement [2]. There can be many reasons for neck discomfort, such as maintaining the same posture for long periods of time [3], lack of sleep [4], and structural cervical lesions [5], etc. The harm to the body that neck discomfort causes does not appear within a short period [6]. If neck discomfort is not resolved, it can cause dizziness [7], nausea [8], irritability [9], shoulder and neck pain [10], and other symptoms. Long-term maintenance of this state is detrimental to physical health and affects the mobility of the entire cervical spine [11]. Cervical range of motion (CROM) [12] refers to the movement of the cervical spine in the sagittal, coronal, and horizontal planes, and it includes forward flexion, extension, left and right lateral flexion, and left and right rotation. Each degree of freedom in the CROM is a good index for evaluating neck movement function because most neck diseases [13, 14] cause changes in cervical spine mobility. Clinically, CROM refers to the range of motion of the cervical spine, the cervical spine’s left and right bends are 45 degrees each, and its left and right rotations are 45 degrees each. A normal cervical spine’s range of motion includes 35 to 45 degrees of forward flexion and 35 to 45 degrees of backward extension [15]. A limited CROM is detrimental to health [16].

There have been many interventions to improve CROM that use a variety of exercise methods [17, 18], and among these is mind-body exercise. Mind-body exercise is a multi-modal exercise [19, 20], and its typical characteristics include slow body movements, whole body stretching and relaxing, breath control, mental concentration, and other structured movements. Its primary forms include Tai Chi, Qigong, Baduanjin, Wuqinxi, and Yijinjing [21]. When compared to other aerobic or resistance exercises [22, 23], the advantages of mind-body exercise include its slow rhythm and stable intensity [19], which are conducive to long-term health development. Moreover, a considerable amount of research has demonstrated that mind-body exercise is beneficial in the treatment of chronic diseases [24, 25]. It is also significant that mind-body exercise does not require the use of sports equipment [26], its learning cost is low, and its safety is high, enabling it to be promoted to a largescale audience [27].

This article aims to expand the sample size of randomized controlled experiments through meta-analysis [28], in order to explore the effect of mind-body exercise interventions on the cervical spine mobility of people with neck discomfort by providing feasibility opinions for treatment guidelines, clinicians, and related groups.

## Methods

### Registration

We conducted this systematic review in accordance with the guidelines for the Preferred Reporting Items for Systematic Reviews and Meta-analyses (S1 Table) [29], and we completed the research registration on the INPLASY platform (Registration Number: INPLASY202140126).

### Eligibility criteria

#### Inclusion criteria

*Types of study*. This study included select randomized controlled trials (RCTs) [30] in peer-reviewed journals.

*Types of participants*. This study included patients with cervical spine discomfort. The age of the population was ≥18 years. In addition, the population showed neck pain, soreness, stiffness, discomfort, restricted mobility, fatigue, shoulder and back pain, dizziness, and other symptoms that did not involve the neck. Other than head discomfort, there were no neck symptoms caused by other diseases.

*Types of intervention*. To be included in this study, the intervention period needed to be greater than or equal to four weeks. Subjects were generally divided according to two types of intervention and study methods: (1) type I: the intervention group is mind-body exercise, and the control group is other physical therapy or no measures, (2) type II: the intervention group is mind-body exercise + other physical therapy, and the control group is other physical therapy. In these studies, the mind-body exercise included Taijiquan, Baduanjin, Qigong, Wuqinxi, and Yijinjing, while the other physical therapy included acupuncture, traction, massage, static-contraction resistance training, and others.

*Types of outcome measures*. This study aimed to evaluate the effect of mind-body exercise on the cervical spine mobility of patients with cervical spine discomfort. When cervical mobility is limited, there is a corresponding decrease in the muscle strength and endurance of the neck muscles and an increase in fatigue, leading to neck discomfort. Since cervical spine mobility is controlled by cervical spine motor function. After a preliminary search of data, we determined that cervical spine mobility is the most effective and direct indicator of cervical spine motor function. It has gradually become the primary measure for evaluating cervical spine function and cervical spine injury [31, 32]. There are many kinds of measurements of cervical spine mobility, including visual inspection [33], tape measures [34], inclinometer measurement [35], electromagnetic motion analysis [36], and other techniques using electronic measuring instruments [37]. In order to consider the convenience and accuracy of actual measurement tools, this study used the following tools: Multi-Cervical Unit (MCU) [38] and CROM for a measure of cervical spine mobility [39]. In addition, as compared with other measuring instruments, CROM and MCU are the most frequently used tools in practical application. In fact, the results from these tools are also often used as a reference for the accuracy of other cervical mobility measurements and have gradually become the primary measure for evaluating cervical spine function and cervical spine injury. They are also important reference indexes for the assessment of the degree of damage, diagnosis and identification of neck diseases, evaluation of curative effects, and performance of prognostic analyses [14].

#### Exclusion criteria

This study’s exclusion criteria included (1) research with incomplete data (i.e., statistics that do not give the mean ± standard deviation for both before and after the experimental intervention); (2) the use of cervical spine mobility measurement tools that were not MCU or CROM; (3) the presence of medical contraindications, such as fractures and local tumors; and (4) patients who were participating in other clinical trials.

### Information sources and searches

Five research databases were used for retrieval in this study: China National Knowledge Infrastructure (from 1979), Web of Science (from 1950), PubMed (from 1965), Cochrane (from 1991), and EBSCO (from 1949) (Date of retrieval: March 10, 2021). In addition, the references for the included literature were searched manually. This study used two sets of keywords: (1) mind-body exercise, Taiji, Taijiquan, Baduanjin, Qigong, Wuqinxi, and Yijinjing; and (2) cervical spondylosis, neck pain, cervical pain, and neck discomfort. The search formula is shown in S1 File. The literature search was carried out by the author (LXH) and by another collaborator (GBH) to ensure accuracy during data retrieval.

### Data collection process

Each article was extracted by two independent researchers (LXH, GBH) and converted into the following standard forms: (1) descriptive statistics that included the first author of the article, the year of publication, and the health of the subjects; diagnostic criteria, sample size, and number of tested subjects; duration of disease, average age, and age range of the tested subjects; training time per session, number of training sessions per week, and total number of training weeks; forms of mind-body exercise and experimental group and control group interventions; and primary measurement results, adverse events, and follow-up; and (2) quantitative data that included random allocations of subjects; the average ± standard deviation of the baseline data of the experimental group and the control group; and the average ± standard deviation of the experimental group and the control group after intervention. Finally, a third researcher (CH) checked and evaluated the effects of interventions after verifying the information to avoid mistakes caused by human error.

### Risk of bias across studies

In order to independently evaluate the methodology of the included studies, two authors (LXH, GBH) used a modified Physiotherapy Evidence Database (PEDro) [40] Scale to evaluate the included literature. The two authors independently evaluated the literature. If they encountered differences, they discussed and resolved their analyses. If they could not reach an agreement on these differences, the third evaluator (CH) was asked to evaluate the issue so they could finally reach an agreement.

### Data synthesis and additional analyses

By using Stata 14.0 software, all the outcome indicators included in the literature were found to be continuous variables. The mean ± standard deviation was selected for statistics. Because the outcome indicators were measured by different tools, Standard Mean Difference (SMD) was selected for the combined effect size, and all the outcome indicators were tested for heterogeneity. For the results of the tests providing the heterogeneity statistics, *p* values and I^2^ were used, and there was no heterogeneity between the studies if *p* >.10, and there was heterogeneity between the studies if *p* <0.10. By comparison, there was a low and acceptable degree of heterogeneity between the studies if I^2^<25%; a low to medium degree of heterogeneity between the studies if 25%<I^2^<50%; a medium to high degree of heterogeneity between the studies if 50%<I^2^<75%; and a high degree of heterogeneity between the studies if I^2^>75%. Subgroup and sensitivity analyses were used to explore the sources of heterogeneity in outcome indicators, combined effect size and publication bias tests were used to calculate the effect size and risk of bias in the publications, and forest and funnel diagrams were drawn.

## Results

### Study selection

**Fig 1:**
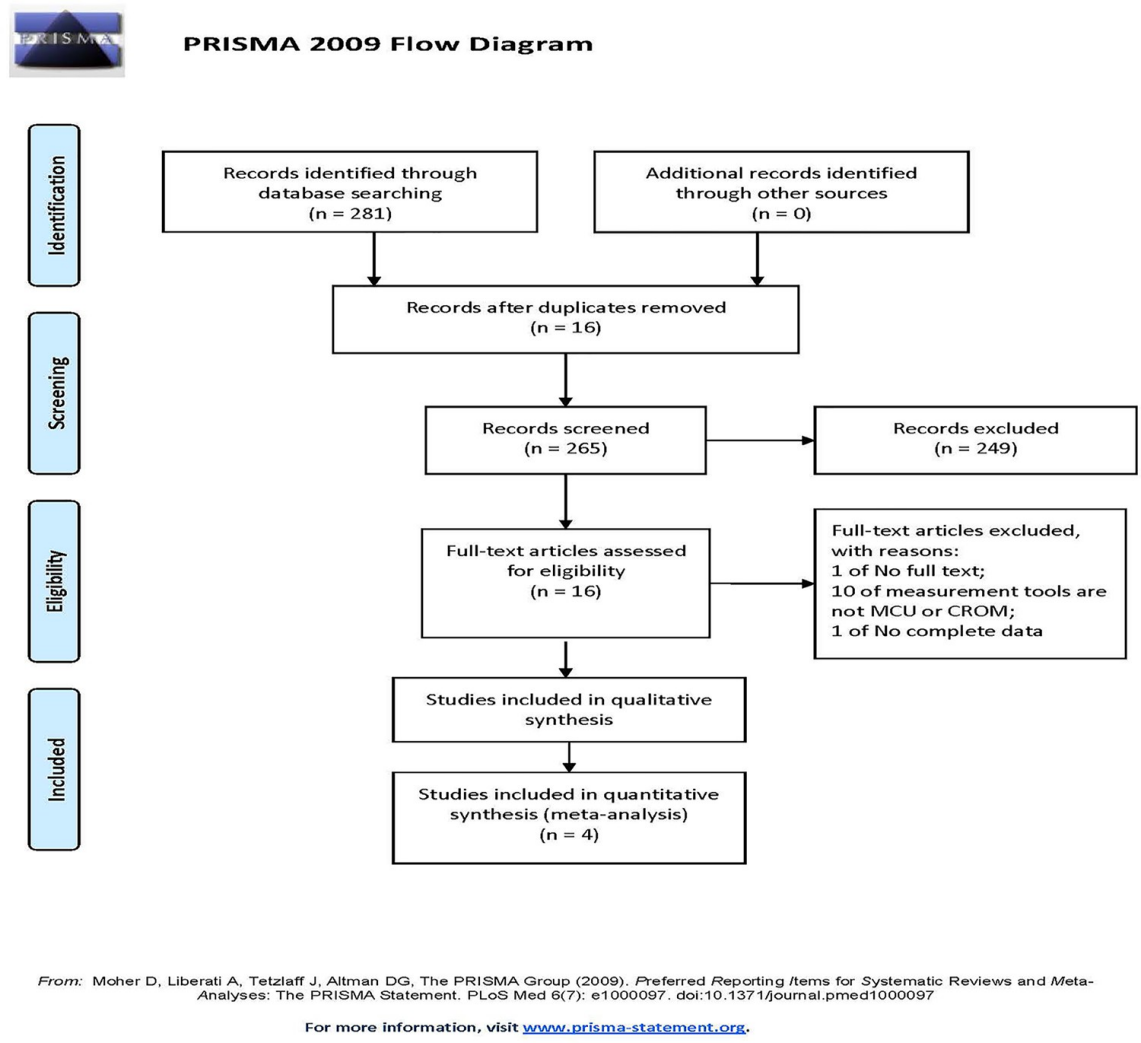
Flow chart depicting exclusion/inclusion of individual studies for meta-analysis.

A total of 281 articles were retrieved through a literature search for this study, and no articles from other sources were added. After deleting 16 duplicate articles, 265 articles remained. The articles were then screened on the basis of their titles and abstracts, and 16 articles were chosen and included in the full-text evaluation. After which, four qualified articles were finally determined and included in the review. Fig 1 outlines the entire article selection process.

### Study characteristics

The publication dates of the articles included in this study are from 2017 to 2020 (one each for 2017, 2018, 2019, and 2020), and all the studies come from China (Table 1). A total of 208 participants are included in this study, and their age range is 18–65 years old. It is worth noting that the age range of the participants in one study is 16–29 years old. The population sub-groups are 168 participants with neck-type cervical spondylosis and 40 participants with neck discomfort. In terms of frequency (weekly) of exercise in the included studies, it is not less than three times per week; in two studies, it is five times, in one study, it is three times, and in one study, it is seven times per week. In terms of time (min) for exercise, it is no less than 20 minutes; in two studies, it is 30 minutes, in one study, it is 30–50 minutes, and in one study, it is 20 minutes. In terms of duration (weeks) of exercise, it is not less than 8 weeks; in two studies, it is 8 weeks, and in two other studies, it is 12 weeks. In terms of type of mind–body exercise, in two studies, it is the first section of the Yijinjing, and in two other studies, it is the complete Baduanjin.

**Table 1:** List of basic characteristics of the included documents.

Author (Year) Country [References]	Subject	Sample	Sex	Course of disease	Age		Training			Intervention	Control	Outcome Measured
					Years	Rang	Frequency (Weekly)	Time (Min)	Duration (Weeks)			
Li (2019) China [41]	NTCS	55	Int (male/female):13/15 Con (male/female):11/16	(m) Int: 9.75 ± 4.64 Con: 10.85 ± 4.92	Int: 36.64 ± 6.45 Con: 36.74 ± 6.50	18~55	5	30	8	Mind-body exercise + Acupuncture (Type I)	Acupuncture	MCU
Huang (2020) China [42]	NTCS	48	/	(m) Int: 6.50 ± 1.60 Con: 5.53 ± 1.69	Int: 38.97 ± 2.37 Con: 40.26 ± 2.09	18~65	5	30	8	Mind-body exercise + Massage (Type II)	Massage	MCU
Xie (2018) China [43]	ND	40	Int (male/female):15/5 Con (male/female):13/7	/	Int: 19.95 (19,21) Con: 19.90 (29,16)	16~29	3	30~50	12	Mind-body excise (Type I)	Static-contraction resistance training	CROM
Shang (2017) China [44]	NTCS	65	Int (male/female):15/20 Con (male/female):11/19	(m) Int: 13.51 ± 3.46 Con: 12.11 ± 4.84	Int: 28.97 ± 10.33 Con:31.01 ± 11.58	25~61	7	20	12	Mind-body exercise + Traction (Type II)	Traction	CROM

Note: Int, Intervention Group; Con, Control Group; NTCS, Neck Type Cervical Spondylosis; ND: Neck Discomfort; MCU: Multi-Cervical Unit; CROM: Cervical Range of Motion; m, months; Type I: the intervention group is mind-body exercise, and the control group is other physical therapy or no measures; Type II: the intervention group is mind-body exercise + other physical therapy, and the control group is other physical therapy.

### Risk of bias within studies

In this study, we used the PEDro scoring scale to score the included articles. When evaluating the articles, we removed Items 5 and 6 from the PEDro scoring scale, providing a full score of 9 points for the PEDro evaluation. The four articles included in this evaluation ranged from 5 to 7 points; Two articles scored 5 points, one article scored 6 points, and one article scored 7 points (Table 2). Therefore, the methodological quality assessment of these articles indicates a medium-to-high quality rating for the included articles, and the results of the quality assessment also reflect the quality of the study.

**Table 2:** Quality assessment of the included studies.

Author (Year) Country [References]	Item 1	Item 2	Item 3	Item 4	Item 5	Item 6	Item 7	Item 8	Item 9	Score
Li (2019) China	1	1	1	1	0	/	1	1	1	7
Huang (2020) China	0	1	/	1	0	/	1	1	1	5
Xie (2018) China	1	1	/	1	0	/	1	1	1	6
Shang (2017) China	0	1	/	1	0	/	1	1	1	5

### Synthesis of results

#### Cervical extension

**Fig 2:**
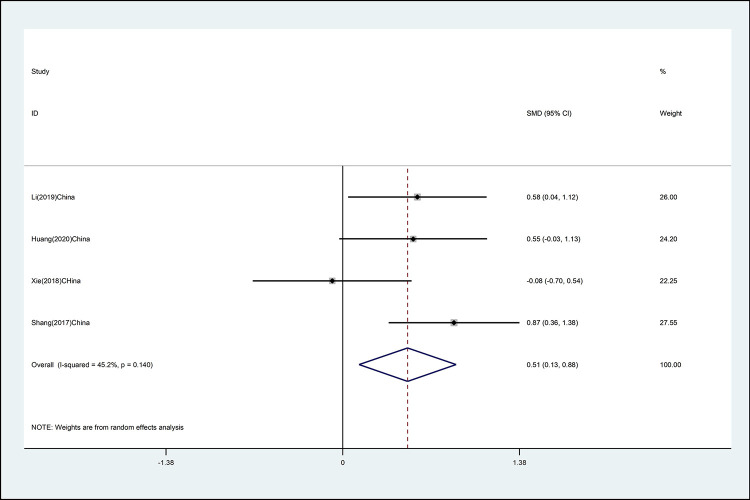
Forest plot of the association between mind–body exercise and cervical extension in overall analysis.

The effects of mind–body exercise on cervical extension is shown in Fig 2 (effect size of [SMD = 0.51 (95% CI 0.13 to 0.88), *p* <0.01; I^2^ = 45.2%]). Mind–body exercise has a significant effect on cervical extension, and there is moderate heterogeneity.

#### Cervical flexion

**Fig 3:**
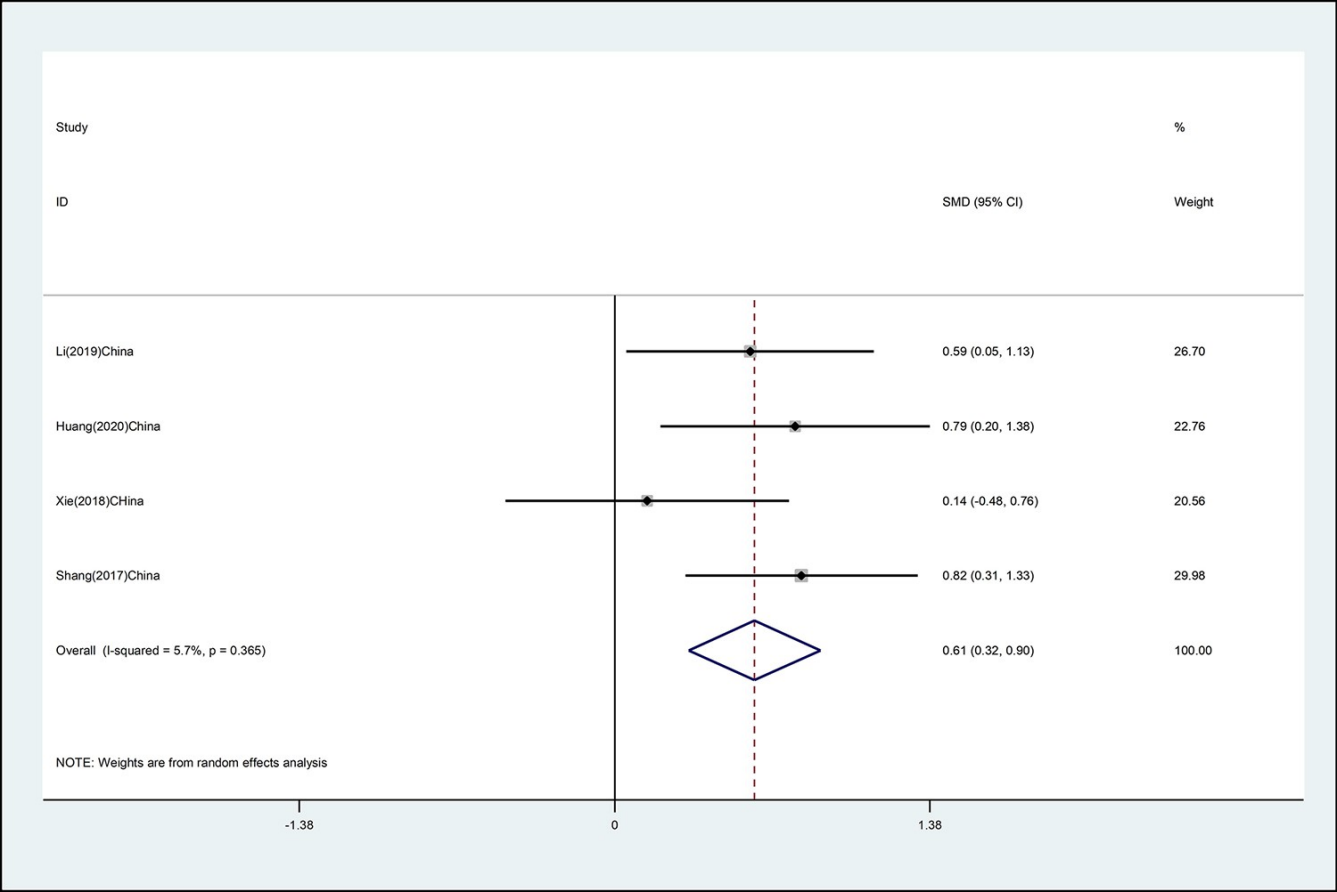
Forest plot of the association between mind–body exercise and cervical flexion in overall analysis.

The effects of mind–body exercise on cervical flexion is shown in Fig 3 (effect size of [SMD = 0.61 (95% CI 0.32 to 0.90), *p* <0.01; I^2^ = 5.7%]). Mind–body exercise has a significant effect on cervical flexion, and there is no heterogeneity.

#### Cervical right lateral flexion

**Fig 4:**
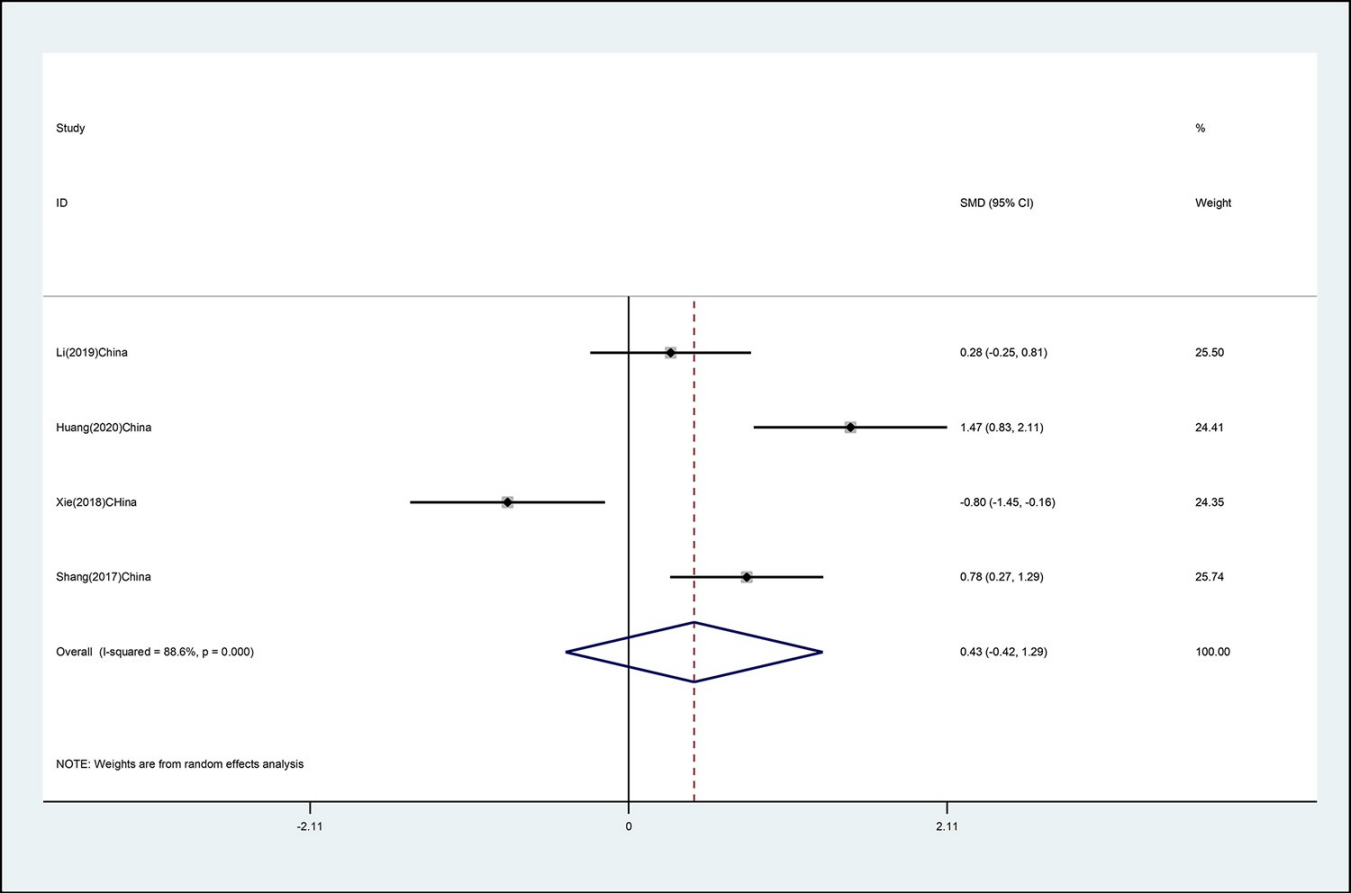
Forest plot of the association between mind–body exercise and cervical right lateral flexion in overall analysis.

The effects of mind–body exercise on cervical right lateral flexion is shown in Fig 4 (effect size of [SMD = 0.43 (95% CI −0.42 to 1.29), *p* >0.05; I^2^ = 88.6%]). Mind–body exercise has no effect on cervical right lateral flexion, and there is high heterogeneity.

#### Cervical right rotation

**Fig 5:**
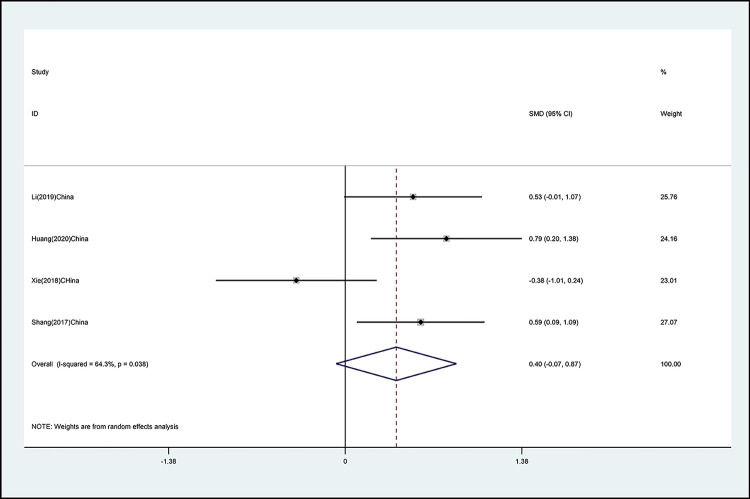
Forest plot of the association between mind–body exercise and cervical right rotation in overall analysis.

The effects of mind–body exercise on cervical right rotation is shown in Fig 5 (effect size of [SMD = 0.40 (95% CI −0.07 to 0.87), *p* >0.05; I^2^ = 64.3%]) Mind–body exercise has no effect on cervical right rotation, and there is medium to high heterogeneity.

#### Cervical left lateral flexion

**Fig 6:**
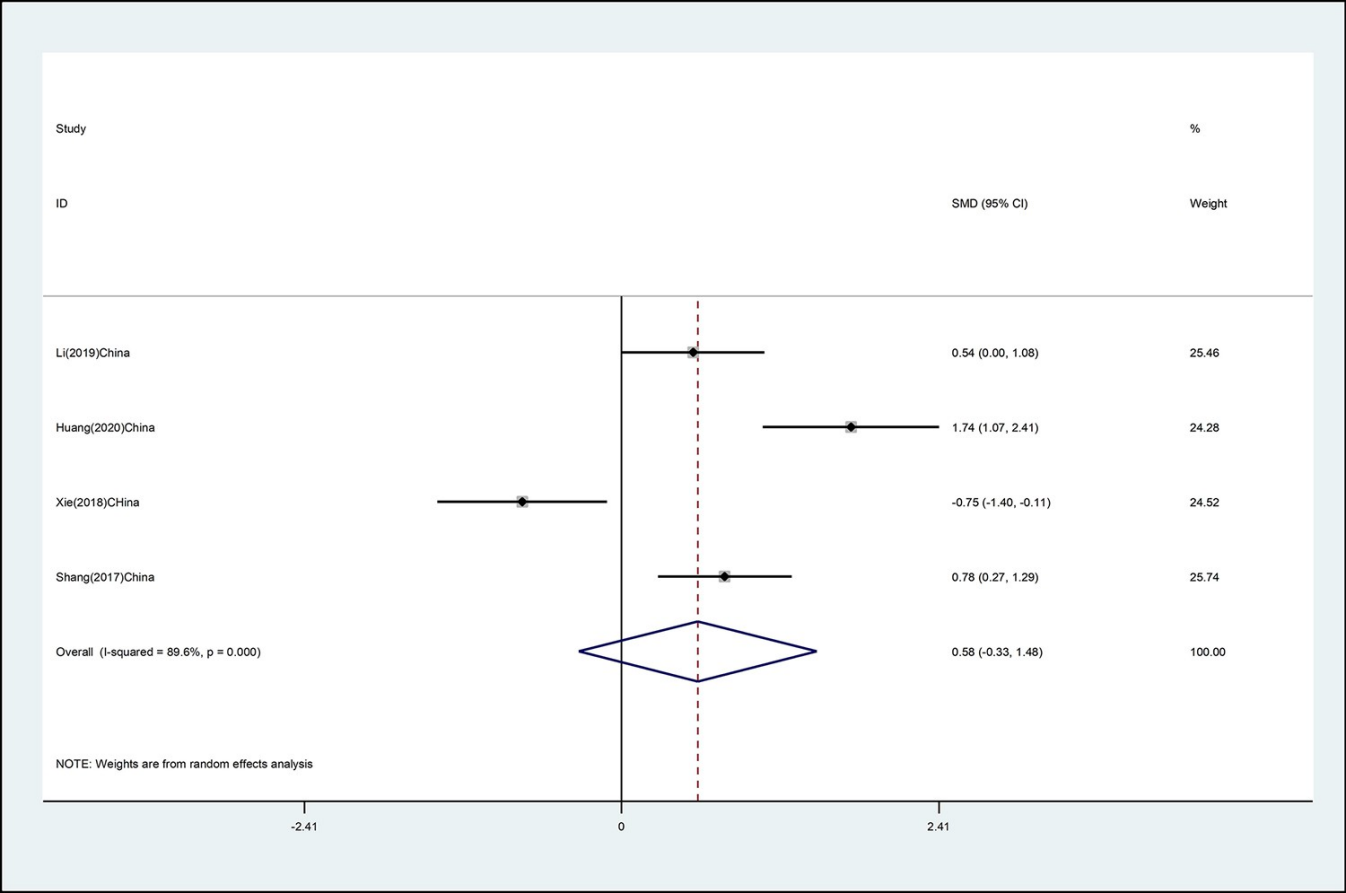
Forest plot of the association between mind–body exercise and cervical left lateral flexion in overall analysis.

The effects of mind–body exercise on cervical left lateral flexion is shown in Fig 6 (effect size of [SMD = 0.58 (95% CI −0.33 to 1.48), *p* >0.05; I^2^ = 89.6%]) Mind–body exercise has no effect on cervical left lateral flexion, and there is high heterogeneity.

#### Cervical left rotation

**Fig 7:**
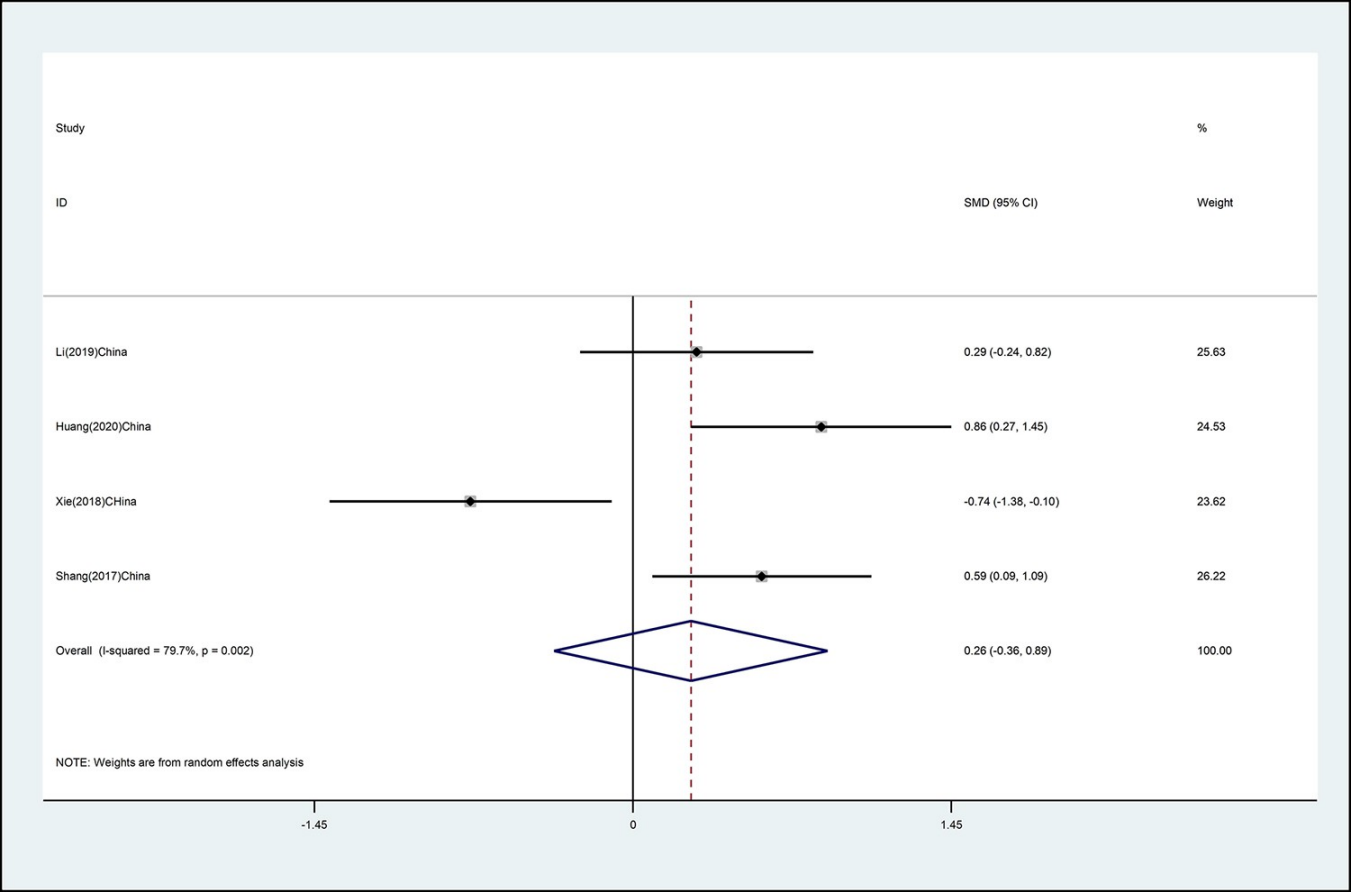
Forest plot of the association between mind–body exercise and cervical left rotation in overall analysis.

The effects of mind–body exercise on cervical left rotation isshown in Fig 7 (effect size of [SMD = 0.26 (95% CI −0.36 to 0.89), *p* >0.05; I^2^ = 79.7%]) Mind–body exercise has no effect on cervical left rotation, and there is high heterogeneity.

#### Risk of bias across studies

**Fig 8:**
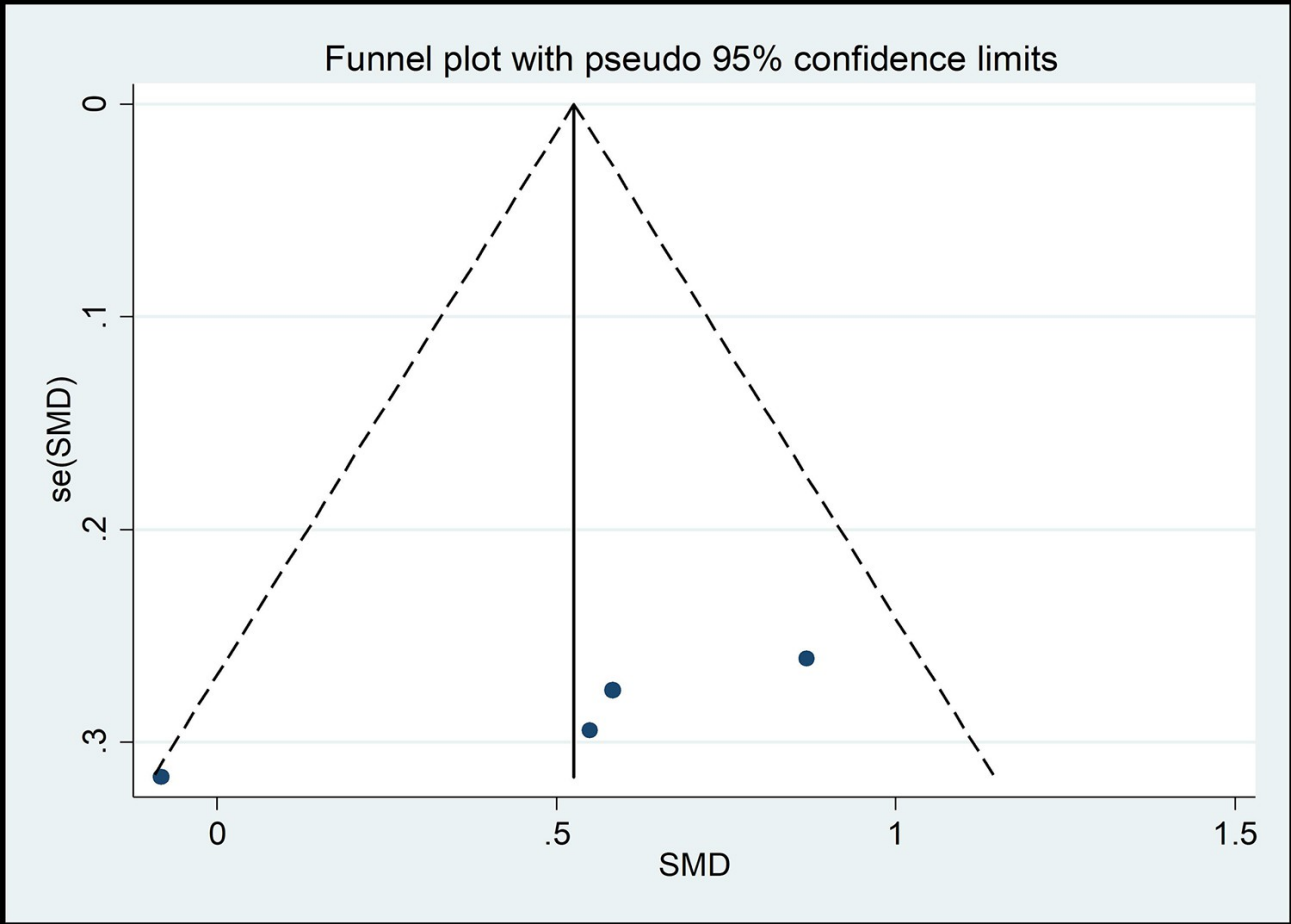
Funnel plot of the association between mind–body exercise and cervical extension.

**Fig 9:**
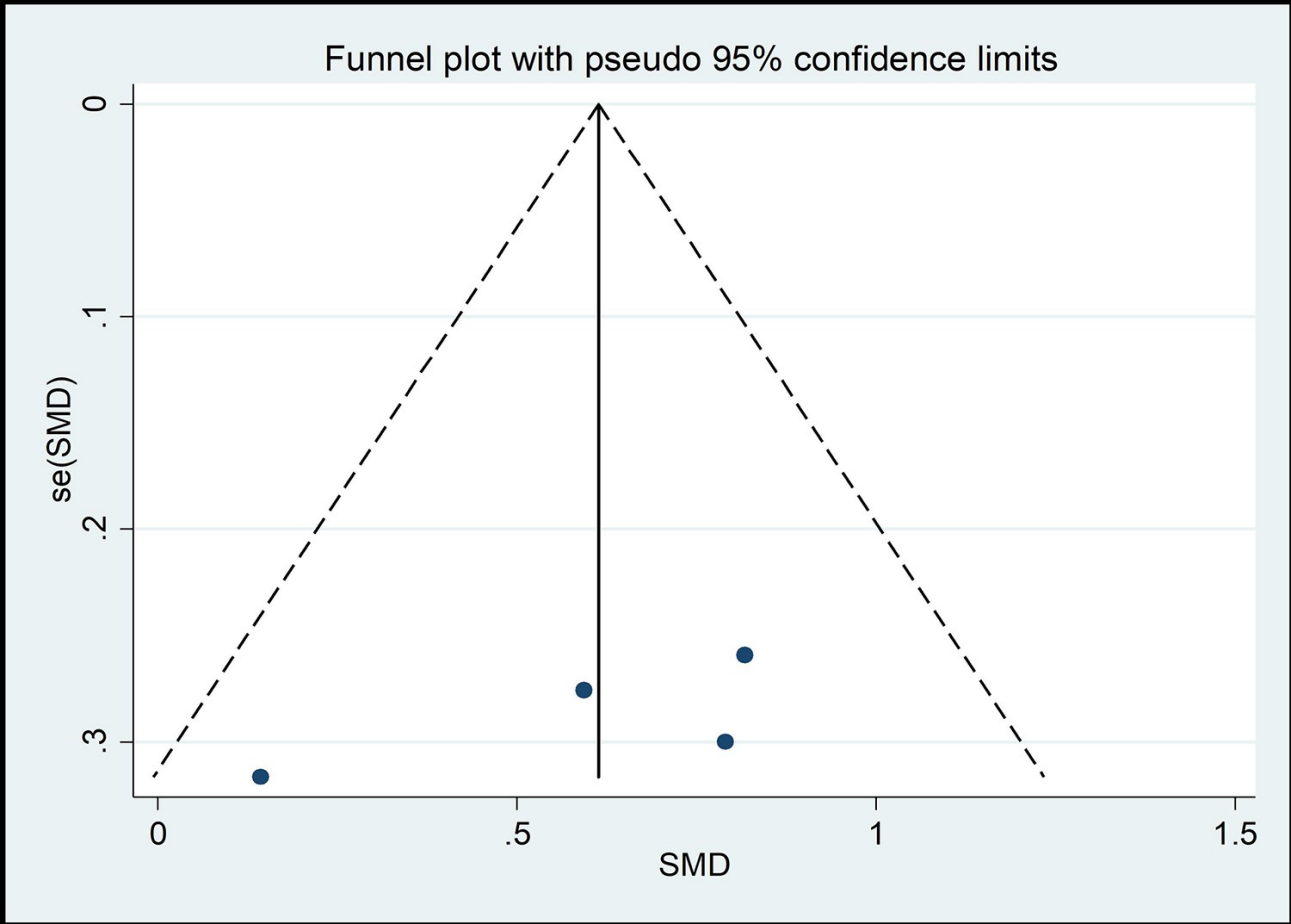
Funnel plot of the association between mind–body exercise and cervical flexion.

**Fig 10:**
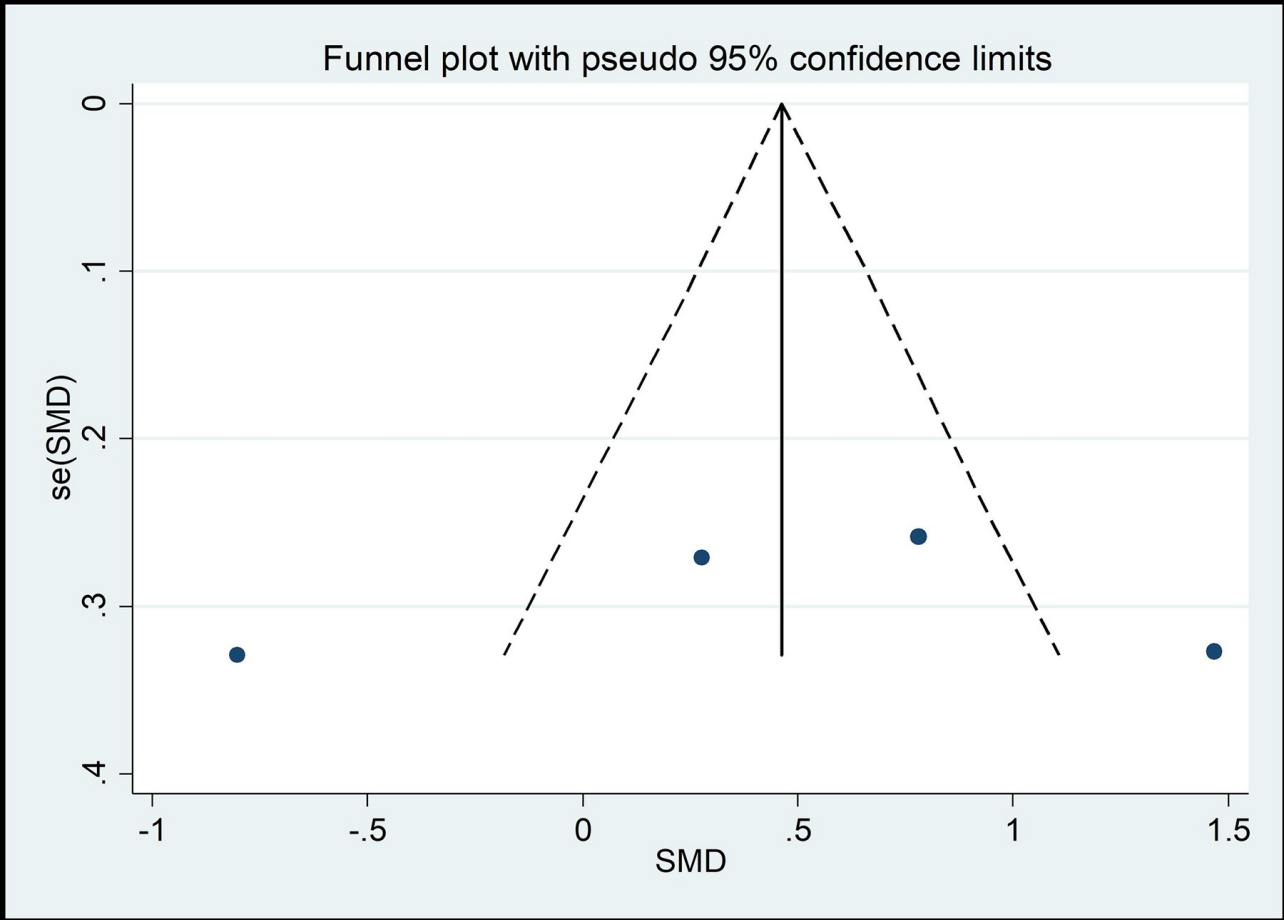
Funnel plot of the association between mind–body exercise and cervical right lateral flexion.

**Fig 11:**
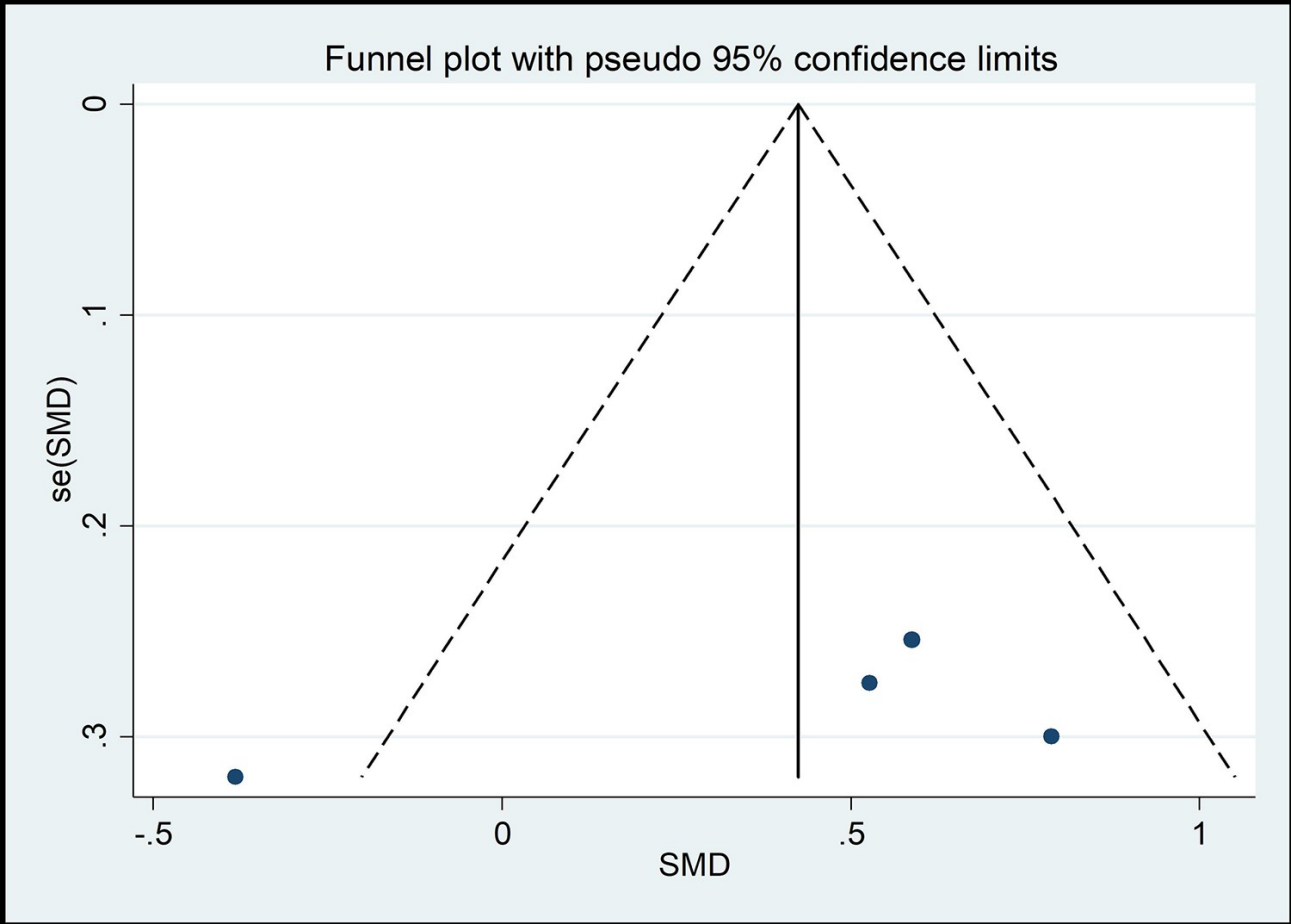
Funnel plot of the association between mind–body exercise and cervical right rotation.

**Fig 12:**
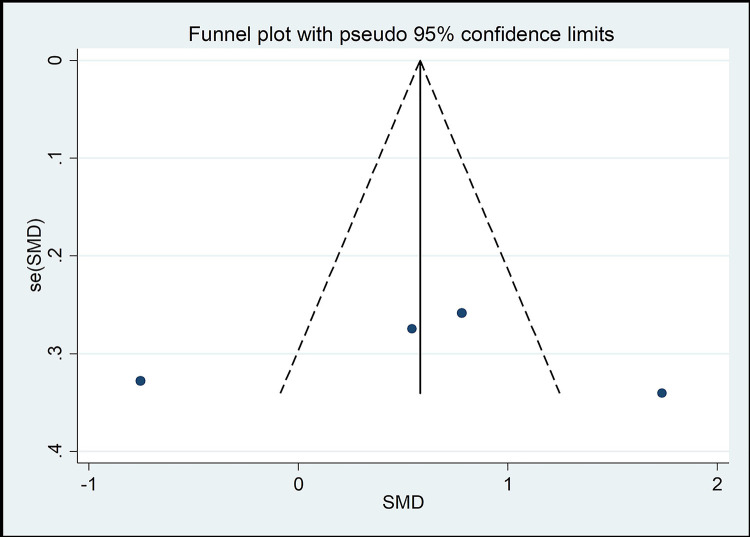
Funnel plot of the association between mind–body exercise and cervical left lateral flexion.

**Fig 13:**
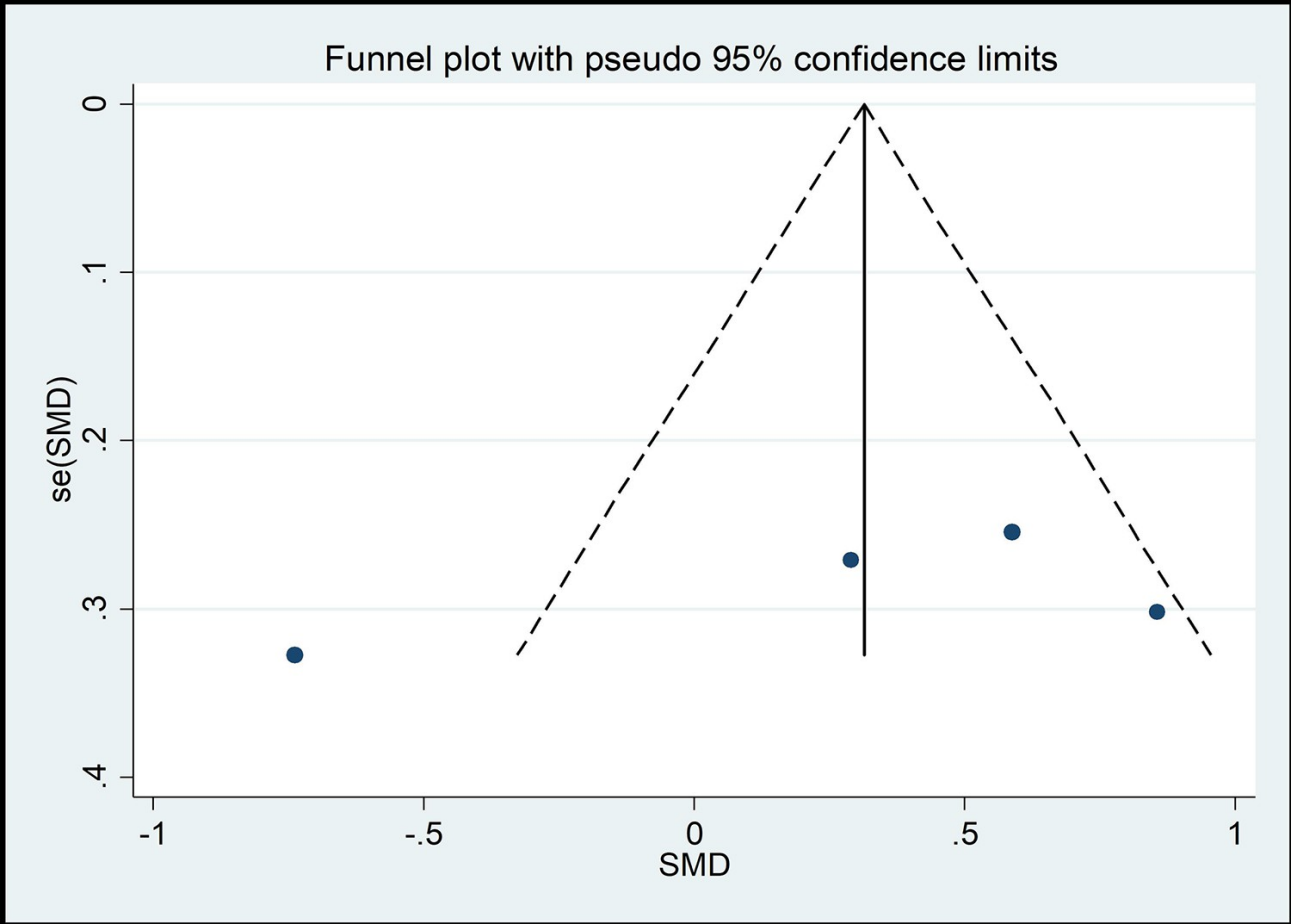
Funnel plot of the association between mind–body exercise and cervical left rotation.

The funnel chart method is the most common method of identifying publication bias [45]. As shown in Figs 8–13, the samples are mainly distributed in the lower part of the funnel and are very discrete. Some samples are outside the confidence interval of the funnel chart, indicating that there is a potential risk of publication bias.

### Additional analyses

#### Subgroup analysis

To determine the source of statistical heterogeneity, the source of heterogeneity is generally considered from the methodological and clinical perspectives (differences in participants, differences in intervention measures, etc.). Methodological perspective: owing to the limited number of studies included in this study, it is not possible to directly grade the research quality. Therefore, a methodological exploration for a source of heterogeneity was not performed. Regarding the clinical perspective, the heterogeneity analysis was not based on differences in intervention measures or outcome indicators because differing degrees of cervical discomfort can make a great impact on the effects of treatment. Instead, since the degree of neck discomfort varies and the cervical discomfort for NTCS patients will often involve other kinds of discomfort besides just neck discomfort, we divided the patients into two groups consisting of an NTCS and neck discomfort group for determining the source of heterogeneity.

As shown in S1–S5 Figs, the cervical extension subgroup analysis result is (NTCS effect size of [SMD = 0.68 (95% CI 0.37 to 0.99), *p*<0.01; I^2^ = 0.0%]), the cervical right lateral flexion subgroup analysis result is (NTCS effect size of [SMD = 0.82 (95% CI 0.18 to 1.46), *p*<0.05; I^2^ = 74.6%]), the cervical right rotation subgroup analysis result is (NTCS effect size of [SMD = 0.62 (95% CI 0.31 to 0.93), *p*<0.01; I^2^ = 0.0%]), the cervical left lateral flexion subgroup analysis result is (NTCS effect size of [SMD = 0.99 (95% CI 0.34 to 1.64), *p*<0.01; I^2^ = 74.8%]), the cervical left rotation subgroup analysis result is (NTCS effect size of [SMD = 0.56 (95% CI 0.25 to 0.87), *p*<0.01; I^2^ = 0.0%]).

#### Sensitivity analysis

To further determine the source of heterogeneity, a sensitivity analysis of the four included studies was conducted, which were numbered 1 [41], 2 [42], 3 [43], and 4 [44]. Sensitivity analysis was conducted on cervical extension, cervical right lateral flexion, cervical right rotation, cervical left lateral flexion, and cervical left rotation, as shown in S6–S10 Figs. Study number 3 [43] is the most sensitive in four studies.

## Discussion

### Summary of evidence

In this review, the research included consists of only RCT studies that used combined effect sizes, subgroup analysis, sensitivity analysis, publication bias tests, and other methods for evaluating the effect of mind-body exercise on the cervical spine mobility of people with neck discomfort.

### Comparisons with previous studies

This study’s search of published data revealed no meta-analyses of studies on the influence of mind-body exercise on the cervical spine mobility of people with neck discomfort. Therefore, we conducted additional searches from the perspective of exercise and found that existing studies have reached a cautious conclusion: yoga can increase the cervical spine mobility of patients with chronic cervical spondylosis, but the specific strength of evidence requires subsequent research and exploration [18]. There is also evidence that stretching exercises can directly affect the range of motion of the cervical spine and that passive hamstring stretching exercises can directly affect the range of motion and balance of the cervical spine [46]. This evidence indicates that exercise may improve the cervical spine mobility of patients with cervical spondylosis.

While there is evidence that exercise may potentially have a positive effect on cervical spine mobility in those experiencing cervical spine discomfort, directly confirmed evidence of the benefits of exercise interventions would more important and useful. Due to the limited number of relevant and existing studies, there is a need to explore related evidence at a later period.

### Potential sources of heterogeneity

To determine the potential sources of heterogeneity, we grouped the participants with neck discomfort through subgroup analysis and found that differences in the diagnoses of the participants were the possible sources of heterogeneity in this study. In addition, a further sensitivity analysis showed that study number 3 [43] was particularly sensitive among the four included studies. Therefore, we have confirmed that differences in the diagnoses of the participants were one of the sources of the heterogeneity. Nevertheless, from the methodological and clinical perspectives, study quality, intervention methods, outcome measures, intervention time, and frequency of exercise may all also be additional sources of heterogeneity. Thus, more research is needed in the future.

### Strengths and limitations

Strengths: First, the results of this study provide support for new and low-cost treatment methods for patients with neck discomfort to improve cervical spine mobility. Second, prior to this study, there had been no studies on treating people with neck discomfort through mind–body exercise, and these results can motivate further follow-up studies on this subject. Third, the research quality scores of the articles included in this study were relatively high and thus the strength of their evidence is also high.

Limitations: First, the sample size of this study is relatively small, and while the obtained results were positive, they may be false positive results. Second, some results have a publication bias, which can reduce the quality of the evidence. Third, the included articles are all in Chinese, and their degree of recognition may not be high.

## Conclusion

This study shows that mind–body exercise has a positive effect on the extension and flexion of people with neck discomfort. However, further research and more reliable evidence are needed to prove that mind–body exercise can be used for the treatment of neck discomfort.

## Supporting information

S1 Fig Forest plot of the association between mind–body exercise and cervical extension in subgroup analysis. (DOCX)

S2 Fig Forest plot of the association between mind–body exercise and cervical right lateral flexion in subgroup analysis. (DOCX)

S3 Fig Forest plot of the association between mind–body exercise and cervical right rotation in subgroup analysis. (DOCX)

S4 Fig Forest plot of the association between mind–body exercise and cervical left lateral flexion in subgroup analysis. (DOCX)

S5 Fig Forest plot of the association between mind–body exercise and cervical left rotation in subgroup analysis. (DOCX)

S6 Fig Sensitivity analysis of the association between mind–body exercise and cervical extension. (DOCX)

S7 Fig Sensitivity analysis of the association between mind–body exercise and cervical right lateral flexion. (DOCX)

S8 Fig Sensitivity analysis of the association between mind–body exercise and cervical right rotation. (DOCX)

S9 Fig Sensitivity analysis of the association between mind–body exercise and cervical left lateral flexion. (DOCX)

S10 Fig Sensitivity analysis of the association between mind–body exercise and cervical left rotation. (DOCX)

S1 Table Preferred reporting items for systematic reviews and meta-analyses (PRISMA) 2009 checklist. (DOC)

S1 File Search strategy. (DOCX)
