# Supplementary material for: The effect of mind-body exercise on cervical spine mobility of people with neck discomfort: A systemic review and meta-analysis of randomised controlled trials
Source: PLoS One. 2022 Jan 21;17(1):e0262429. doi: 10.1371/journal.pone.0262429 (PMC8782300; doi:10.1371/journal.pone.0262429)
Supplement: S9 Fig — (DOCX) [file pone.0262429.s009.docx]

**S9 Fig**


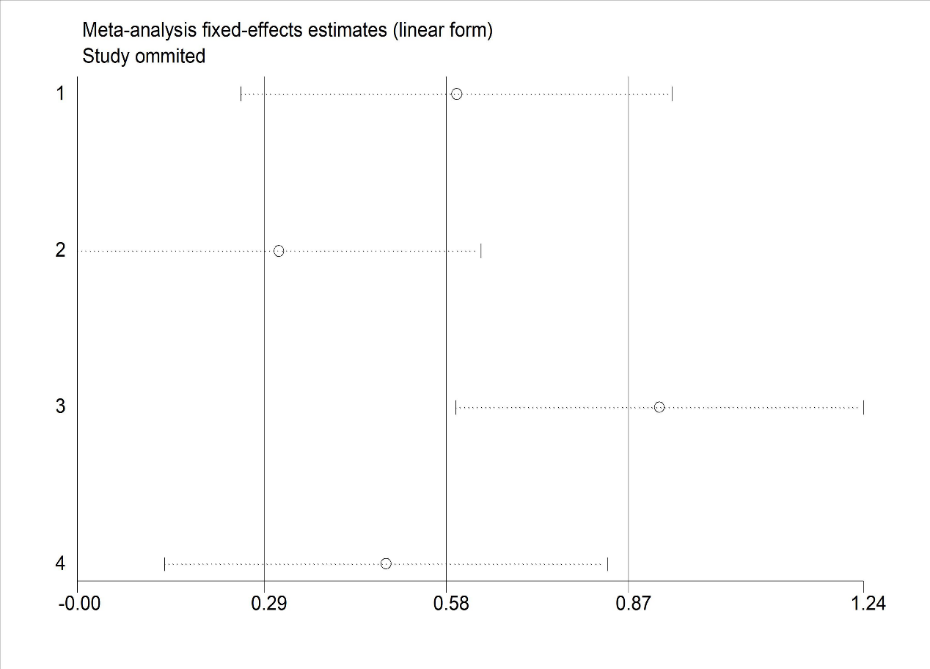


**Sensitivity analysis of the association between Mind–body exercise and Cervical Left Lateral Flexion**
