# Supplementary material for: The effect of mind-body exercise on cervical spine mobility of people with neck discomfort: A systemic review and meta-analysis of randomised controlled trials
Source: PLoS One. 2022 Jan 21;17(1):e0262429. doi: 10.1371/journal.pone.0262429 (PMC8782300; doi:10.1371/journal.pone.0262429)
Supplement: S1 File — (DOCX) [file pone.0262429.s012.docx]

**S1 File. Search Strategy**

**Pubmed**

(neck pain[Mesh Terms] OR cervical spondylosis[Mesh Terms] OR cervical pain[Mesh Terms] OR neck discomfort[Mesh Terms]) AND (mind-body exercise[Mesh Terms] OR taichi[Mesh Terms] OR taijiquan[Mesh Terms] OR baduanjin[Mesh Terms] OR qigong[Mesh Terms] OR wuqinxi[Mesh Terms] OR yijinjing[Mesh Terms]）

Form 1965

**Cochrane library**

#1: neck pain

#2: cervical spondylosis

#3: cervical pain

#4: neck discomfort

#5: mind-body exercise

#6: taichi

#7: taijiquan

#8: baduanjin

#9: wuqinxi

#10: yijinjing

#11: qigong

(#1 OR #2 OR #3 OR #4) AND (#5 OR #6 OR #7 OR #8 OR #9 OR #10 OR #11)

From 1991

**WOS (Web of science)**

TS = (neck pain OR cervical spondylosis OR cervical pain OR neck discomfort) AND TS = (mind-body exercise OR taichi OR taijiquan OR baduanjin OR qigong OR wuqinxi OR yijinjing)

From 1950

**EBSCO**

SU = (neck pain OR cervical spondylosis OR cervical pain OR neck discomfort) AND SU = (mind-body exercise OR taichi OR taijiquan OR baduanjin OR qigong OR wuqinxi OR yijinjing)

From 1949

**CNKI（China National Knowledge Infrastructure）**

SU = neck pain + cervical spondylosis + cervical pain + neck discomfort AND SU = mind-body exercise + taichi + taijiquan + baduanjin + qigong + wuqinxi + yijinjing

Form 1979
